# Does social support reduce bodily pain among African American women with SLE? Findings from a randomised controlled trial

**DOI:** 10.1136/lupus-2025-001712

**Published:** 2025-10-20

**Authors:** Jerik Leung, Everette P Keller, Paul Nietert, Tomika Caldwell, Clara L Dismuke-Greer, Hetlena Johnson, Edith Williams

**Affiliations:** 1 Behavioral, Social, and Health Education Sciences, Emory University, Atlanta, Georgia, USA; 2 Public Health Sciences, Medical University of South Carolina, Charleston, South Carolina, USA; 3 Health Economic Resource Center, VA Palo Alto Health Care System, Palo Alto, California, USA; 4 Lupus Community Advocate/Researcher, Charleston, South Carolina, USA; 5 Public Health Sciences, University of Rochester Medical Center, Rochester, New York, USA

**Keywords:** Lupus Erythematosus, Systemic, Pain, Quality of LIfe, Psychology, Health-Related Quality Of Life

## Abstract

**Objective:**

Peer mentoring has been shown to be an effective intervention for chronic conditions with evidence to suggest that it might improve health-related bodily pain among African American women living with systemic lupus erythematosus (SLE). However, there is a lack of evidence to describe the intervention impact when adjusting for self-management of SLE. The present work aims to determine whether greater patient activation is associated with greater reductions in pain overall and within intervention groups.

**Methods:**

Data were used from the Peer Approaches to Lupus Self-Management study, a randomised controlled trial designed to determine the efficacy of peer mentorship in African American women with SLE. A total of 274 participants were randomised to an intervention (mentorship) or control (non-mentorship) arms. Data were collected on self-reported Lupus Quality of Life questionnaire for bodily pain and Patient Activation Measure (PAM). Linear mixed models and multivariable linear mixed models were fit to assess the intervention and impact of PAM on bodily pain over time.

**Results:**

Increased patient activation was significantly associated with greater reductions in bodily pain (b=−0.13, p=0.019); however, there was no significant difference in intervention group over the study period between the intervention and control groups

**Conclusion:**

Patient self-management can have a significant effect on bodily pain for SLE patients. Future work aims to consider strategies which address patient activation as a mechanism for reducing pain and improving quality of life.

**Trial registration number:**

NCT03734055.

**WHAT IS ALREADY KNOWN ON THIS TOPIC**

- Peer-mentoring interventions which deliver social support have been shown to be effective for addressing impaired quality of life among people living with chronic disease but are underexplored among those living with systemic lupus erythematosus.

**WHAT THIS STUDY ADDS**

- Patient activation, or the skills, knowledge, and confidence to manage one’s health condition, was associated with reduced bodily pain over time for both experimental and control groups.

**HOW THIS STUDY MIGHT AFFECT RESEARCH, PRACTICE OR POLICY**

- Pain is one of the most common symptoms of systemic lupus erythematosus and this study provides valuable mechanistic knowledge for future work to understand how patient activation and social support relate to pain and then consequently how to leverage those mechanisms to reduce pain.

## Introduction

 Chronic pain is one of the most frequently reported symptoms of people living with systemic lupus erythematosus (SLE).^1 2^ A recent retrospective study from a California electronic health record (EHR) system suggested that 61% of patients with established care reported a chronic overlapping pain condition.^3^ Researchers in a separate EHR study focusing on emergency department (ED) visits among people with SLE similarly found that pain was the chief complaint in the majority of visits among frequent ED users, of whom were more likely to be African American and come from socioeconomically deprived neighbourhoods.^4^ In a disease notorious for injecting uncertainty into the patient experience,^5^ the prevalence of pain is a unifier in characterising the impact of SLE on daily living.

When considering pain as a symptom of SLE, it is important to acknowledge the context of the sociodemographic groups most burdened by this disease. Pain as an unmet need disproportionately affects women of colour, particularly black women, given the disproportionality in prevalence of SLE,^6^,^9^ generally worse outcomes, worse comorbidities^10^ and worse health-related quality of life (HRQOL) relative to the general population with SLE.^11 12^ These data points on pain prevalence, in conjunction with the disparities present in the SLE population, suggest that interventions must attend to the ubiquity of pain and the sociocultural context of the groups most burdened by disease.

Recent conceptual work in pain research provides general and SLE-specific frameworks for thinking about pain. In 2016, a collaboration of pain researchers put forth a framework, which included three categories of pain: nociceptive (pain originating from tissue damage), neuropathic (pain originating in a peripheral or central nerve) and nociplastic (pain resulting from altered nociceptive processing).^13 14^ Importantly, much of the pain characterising the experience of people with SLE may be nociplastic in origin. Symptoms which may be categorised under nociplastic pain include body-wide pain, fibromyalgia and may also be related to non-pain symptoms such as fatigue, brain fog or even feelings of helplessness/overwhelming. This is problematic since, as noted by Murphy *et al*, ‘rheumatologists assume that all pain is nociceptive in origin in rheumatic disease’ but lack the training/tools to treat such nociplastic pain.^13^

An additional framework for pain specific to SLE, primarily for clinical categorisation, has been the type 1/type 2 symptom paradigm.^15^ Type 1 symptoms are considered classical signs of SLE that have clear physiological autoimmunity and respond to biomedical treatments. Type 2 symptoms are aspects of SLE that have an unclear linkage with physiological autoimmunity (ie, do not necessarily correlate with disease activity).^15^ Type 2 symptoms are highly present in evidence from patient perspectives.^16^ Additionally, type 2 symptoms may respond to a variety of methods of intervention and generalised pain would be classified as type 2.^17^

The presence of these pain frameworks is important for SLE because discordance between patients and providers is a critical barrier among patients with SLE.^18^ Implications of these frameworks collectively suggest the need for development and testing of non-pharmacologic interventions due to their potential to more sufficiently modify nociplastic pain, along with other type 2 symptoms (eg, brain fog, chronic fatigue). These frameworks may help to establish expectations of what treatments target which symptoms for patients, meaning that biomedical interventions target neuropathic pain but may not have strong efficacy in nociplastic pain.

Evidence which is framed outside of the typical biomedical frame suggests strategies, which may target pain and other aspects of HRQOL. In an examination of pain outcomes among a prospective SLE cohort, Falasinnu *et al* suggested that disease activity and organ damage only account for 33% of variance in pain and that this relationship is attenuated when accounting for a variety of sociodemographic and psychosocial factors.^1^ This finding, along with other work, suggests the critical role of social and psychosocial factors in reducing (or exacerbating) the impact of SLE on patient experience.^11^ In general, non-pharmacologic interventions are a critical piece of how to improve HRQOL for people living with SLE due to their role in enhancing and complementing biomedical focused interventions.^19^ Moreover, a framework around equity is necessary due to the documented disparities in populations burdened by SLE, population-wide disparities.^6^,^9^ These data suggest that non-pharmacologic interventions that attend to the psychosocial factors most specific to are needed.

Peer-mentoring interventions are a type of evidence-based self-management intervention that are designed to deliver a variety of intervention content and health education and social support.^20 21^ A key strategy is that intervention participants receive this content from someone viewed as a peer, often matching on certain characteristics (eg, similar sociodemographics, living in the same neighbourhood, living with the same illness). These interventions are suitable for SLE because social support is a key unmet expressed need.^22^ Additionally, social support has been linked to a variety of outcomes of SLE and may be related to reduction in pain.^23^,^27^ These prior studies offer promise for social support interventions, have the potential to reduce inequities and may be linked to improvements in HRQOL.

The present study reports pain-focused findings of the Peer Approaches to Lupus Self-management (PALS) study, a randomised controlled trial (RCT) testing a peer support intervention to improve quality of life among black women living with SLE. The objective of this study was to evaluate the impact of the intervention on bodily pain, and the extent to which patient activation, conceptualised as skills, knowledge, and confidence to manage one’s health condition, modifies this relationship.

## Patients and methods

### PALS overview

The PALS study is a culturally tailored peer-support intervention study that primarily focuses on improving disease self-management, disease activity and HRQOL among African American women with SLE. Under a core intervention strategy of peer-support, participants were recruited as peer mentors and mentees.^21^ Mentors were paired with up to three mentees and delivered intervention content through twelve 60 min virtual (telephone or video-call) sessions over 24 weeks. Participants focused on a different topic each session and topics included those adapted from the Chronic Disease Self-Management Program^28^ (eg, Goal Setting, Action-planning, exercise, effective communication, the Arthritis Self-Management Program^29^ and the Systemic Lupus Erythematosus Self-Help Course).^30^ Additional included sessions were influenced from formative work on unmet needs of African American Women with SLE (eg, body image, self-monitoring, sexuality/sexual health, trust).^2 22 31 32^ Mentors were trained in strategies salient to delivery of the planned content such as conversational approaches to facilitate session objectives without being prescriptive, and mentors were instructed not to provide clinical advice.

### Recruitment and sampling

The PALS study was approved by the Medical University of South Carolina Institutional Review Board (IRB protocol # Pro00080875). Women with active SLE who self-identified as African American were recruited using a lupus patient database housed at Medical University of South Carolina from 1 December 2018 to 30 November 2021, with both mentors and mentees being recruited through this database.^33^ Inclusion criteria for mentees and mentors included: (1) African American race and female gender; (2) clinical diagnosis of SLE from a physician, according to ACR revised criteria for SLE; and (3) 18 years of age or older (13). Additional inclusion criteria for mentors included: (1) disease duration >2 years; (2) able to attend scheduled training sessions; and (3) willing to provide one-on-one support to up to three African American women with SLE. Mentors were screened for competence, maturity, emotional stability and verbal communication using the following scales: the Arthritis Impact Measurement Scales, the Arthritis Helplessness Index, Wallston General Perceived Competence Scale, University of California at Los Angeles Loneliness Scale, Rosenberg Self-Esteem, Campbell Personal Competence Index, Carkhuff Communication and Discrimination Skills Inventory and the Applied Knowledge Assessment scales.^34^ The study project coordinator who enrolled participants was blinded to participant assignment.

### Study design

The PALS study used a randomised controlled design with intervention and control arms (ClinicalTrials.gov # NCT03734055). Mentees were randomised into either intervention or control groups using block randomisation to ensure equal sample size between treatment arms.^35^ Using a block size of three, participants were assigned to the appropriate treatment condition as they enrolled in the study until the block was completed. Then the following three participants were assigned based on the next block. This method was repeated until 100 participants were reached per wave of the study. There were three waves totalling 316 participants enrolled.

**Figure 1:**
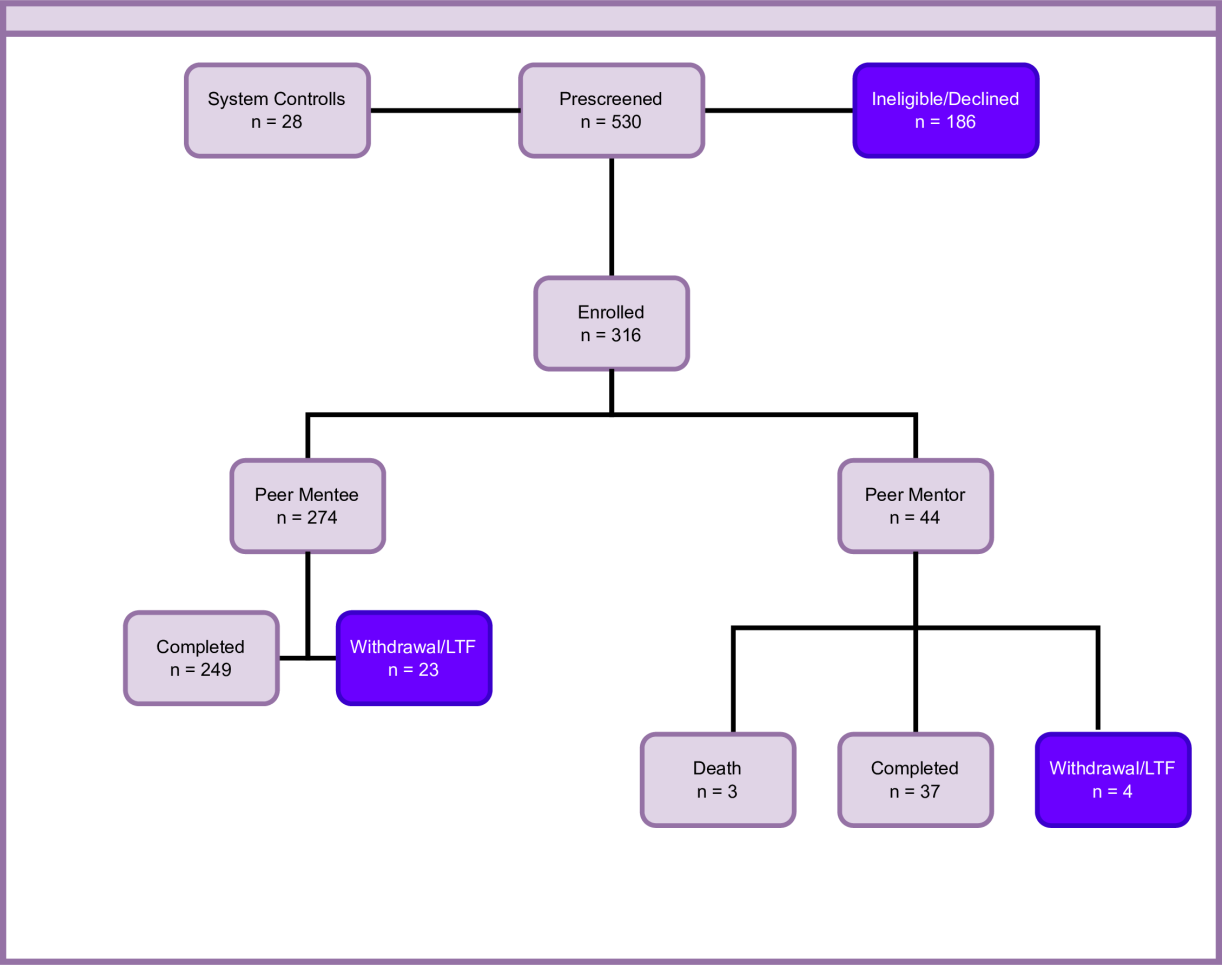
PALS consort diagram. LTF, loss to follow up; PALS, Peer Approaches to Lupus Self-Management.

The intervention was delivered in three waves with mentees and mentors split approximately evenly in each wave. After recruitment, 274 mentees and 44 mentors began the intervention (figure 1). Mentors were not allocated to the control arm and mentees remained in their assigned group for the duration of the trial. Mentees randomised into the control arm were enrolled in a support group only open to participants in this trial and moderated by study team members. Mentees in the control arm support group followed the same schedule (bi-weekly) as those in the experimental arm. Mentors were trained to deliver intervention content, prior to being paired up with up to three mentees. The principal roles of the peer mentors were to: (1) provide information about SLE, SLE-related behaviours, thoughts and feelings, and the nature of recommended treatments; (2) provide social support to alleviate the mentee’s sense of social isolation; (3) enhance and reinforce the mentee’s sense of self-efficacy to manage the condition and (4) encourage the mentee to participate actively in the recommended self-management skills building therapy.

 The PALS study was powered based on the primary outcome in change of lupus HRQOL outcomes between baseline and 12-month post intervention. Assuming three measurement time points, level of significance α=0.05, two-tailed comparison, correlation between pairs of measurements within participants (interclass correlation) no larger than ρ=0.6, and compound symmetry covariance structure, a sample size of 123 participants per group (total 246) was needed to detect a standardised effect size of at least 0.35 SD with 80% power, which included a 20% inflation to account for study attrition.

### Data collection and measures

Assessments were administered as a REDCap survey at baseline, midpoint (3 months from baseline), postintervention (6 months from baseline) and 12 months from baseline. All participants (intervention, control and peer mentors) were assessed on the same schedule, using the same measures. The primary outcome for bodily pain was assessed using the bodily pain subdomain of the Lupus Quality of Life Questionnaire (LUP-QOL BP).^36 37^ This measure was normalised to scale between 0 and 100. Higher score corresponds to greater pain (lower HRQOL). Patient activation was assessed using the Patient Activation Measure (PAM)^38^ where a higher PAM score suggests greater activation. The trial ended on the originally planned schedule.

### Statistical approach

Demographics and questionnaire results were summarised for the experimental and control arms of study participants. For normally distributed variables, means and SD were calculated for each group. For categorical variables, proportions were calculated for each categorical subset in the respective treatment group.

The primary outcome of interest was the LUP-QOL BP measure captured at baseline and at 3 months, 6 months and 12 months postintervention. Linear mixed models were fit to include a random subjects effect to account for the natural within-subject correlation of LUP-QOL BP measures captured from the repeated measures over the duration of the study. Multivariable linear mixed models were fit to examine the association between PAM and each predictor, which included effects for visit, intervention group, a visit-by-intervention group interaction, the baseline LUP-QOL BP measure, PAM measure, a participant’s age (described in years as: <25, 25–34, 35–44, 45–54, 55–64 and ≥65), level of education (defined by a participant’s highest level of education achieved, categorised as: non-high school graduate, high school graduate, some college and college graduate), whether the participant was married, and whether the participant had insurance. Statistical assumptions were assessed graphically with transformations performed as necessary. P values <0.05 were considered statistically significant. All statistical analyses were performed using SAS Studio.

### Patient and public involvement

Individuals living with SLE were involved in this research as both participants and as collaborators in the development of PALS. The PALS team conducted pilot testing of intervention materials with participants prior to the final intervention described in this manuscript. During the pilot phase, we asked participants to asses thes burden of the intervention and provide feedback on how to provide content differently or more concisely. Additionally, patients with SLE were critical fo ther recruitment process as we asked individuals to refer people within their networks to the PALS study, in addition to recruitment through our clinic and community networks. In addition, patients with SLE served as the interventionists and were trained to deliver self-management content to their peers. Finally, patients with SLE were involved in the dissemination of this research, as evidenced by our authorship team.

## Results

The demographic characteristics of participants are summarised in table 1. Of the 274 participants, 137 (50%) were assigned to the control arm and 137 (50%) were assigned to the experimental arm. In general, the groups were comparable with respect to demographics: age, highest level of education achieved, marriage status and insurance status.

**Table 1:** Summary of PALS 1 data: demographics

	Overall	Control	Intervention
*Number of subjects (overall*)	274	137 (50%)	137 (50%)
*Number of subjects across visits: n (%*)			
*Baseline*	273	136 (49.82%)	137 (50.18%)
*3* months	246	125 (50.81%)	121 (49.19%)
*6* months	243	124 (51.03%)	119 (48.97%)
*12* months	228	118 (51.75%)	110 (48.25%)
*PAM score across visits: mean (SD*)			
*Baseline*	74.55 (15.09)	74.85 (16.44)	74.27 (13.77)
*3* months	74.51 (15.25)	73.84 (15.16)	75.20 (15.38)
*6* months	76.31 (13.65)	76.91 (14.17)	75.69 (13.13)
*12* months	76.48 (14.80)	76.18 (14.47)	76.80 (15.21)
*Age*			
*24 or less*	27 (10.15%)	12 (9.30%)	15 (10.95%)
*25–34*	68 (25.56%)	33 (25.58%)	35 (25.55%)
*35–44*	70 (26.32%)	29 (22.48%)	41 (29.93%)
*45–54*	50 (18.80%)	30 (23.26%)	20 (14.60%)
*55–64*	43 (16.17%)	22 (17.05%)	21 (15.33%)
*65 or more*	8 (3.01%)	3 (2.33%)	5 (3.65%)
*Education*			
*Less than HS*	25 (9.43%)	12 (9.30%)	13 (9.56%)
*HS graduate*	51 (19.25%)	21 (16.28%)	30 (22.06%)
*Some college*	88 (33.21%)	38 (29.46%)	50 (36.76%)
*College graduate*	101 (38.11%)	58 (44.96%)	43 (31.62%)
*Marriage*			
*Married*	64 (24.15%)	30 (23.44%)	34 (24.82%)
*Insurance*			
*Yes*	246 (93.18%)	121 (95.28%)	125 (91.24%)

HS, high school; PALS, Peer Approaches to Lupus Self-Management; PAM, Patient Activation Measure.

Study outcomes comparing whether any significant association exists between PAM measures adjusting for PAM score, visit, treatment, a treatment-by-visit interaction, baseline LUP-QOL BP measure, age, level of education, marital status and insurance are summarised in table 2. Baseline LUP-QOL BP scores were significantly (p<0.001) associated with changes in LUP-QOL BP scores, for both intervention and control groups. Moreover, greater PAM score was associated with decreased pain (b=−0.13, p=0.019). No additional variables were significantly (p<0.05) associated with LUP-QOL bodily pain.

**Table 2:** Summary of fixed effects

Predictor	Estimate (95% CI)	Type III p value
*Intercept*	9.841 (−3.791 to 23.474)	0.156
*Baseline LUP-QOL BP*		
*Intervention*	−0.283 (−0.400 to 0.166)	<0.001
*Control*	−0.328 (−0.435 to 0.222)	<0.001
*Treatment*	−1.639 (−9.995 to 6.717)	0.727
*Visit*		0.879
*Post: 6* months	1.199 (−3.900 to 6.298)	
*Post: 12* months	−0.291 (−4.174 to 3.592)	
*Treatment×Visit*		0.769
*Intervention×Post (6 months*)	1.199 (−3.900 to 6.298)	
*Intervention×Post (12 months*)	−0.664 (−6.210 to 4.880)	
*PAM Score*	−0.13 (−0.238 to −0.021)	0.019
*Age*		0.688
*≤ 24*	Ref	
*25–34*	5.890 (−1.939 to 13.719)	
*35–44*	5.039 (−2.864 to 12.941)	
*45–54*	6.685 (−1.625 to 14.996)	
*55–64*	4.814 (−3.639 to 13.267)	
*≥ 65*	1.300 (−12.045 to 14.644)	
*Education*		0.324
*<HS*	Ref	
*HS graduate*	5.404 (−2.827 to 13.635)	
*Some college*	1.431 (−6.236 to 9.098)	
*College graduate*	0.178 (−7.360 to 7.715)	
*Married (vs non-married*)	−2.392 (−7.173 to 2.390)	0.325
*Insured (vs non-insured*)	−1.497 (−10.037 to 7.043)	0.730

HS, high school; LUP-QOL BP, Lupus Quality of Life Questionnaire.

Examination of the study outcome over time is summarised in table 3 for the intervention and control groups at the time points of interest and by 25-point increments in PAM score. Participants with self-reported PAM scores greater than 50 units had significant (p<0.05) decreases (ie, improvements) in estimated LUP-QOL BP scores from baseline in the intervention and control groups. Moreover, participants with a self-reported PAM score of 50 units had significant (p<0.05) decreases in estimated LUP-QOL BP scores from baseline to 6 months (mean difference from baseline: −8.9; 95% CI −17.02 to –0.83; p=0.031) in the control group and from baseline to 3 months (mean difference from baseline: −7.79; 95% CI −15.56 to 0.02; p=0.049) as well as 12 months (mean difference from baseline: −8.75; 95% CI −16.83 to 0.66; p+0.034) in the intervention group. There were no significant between-group differences observed at any time points. There were no harms or unintended effects to report for any group in this trial.

**Table 3:** Summary of changes in LUP-QOL bodily pain measures over time by PAM measure

LUP-QOL changes over time	Control	Intervention	Control vs intervention
*PAM score=0*			
*Baseline to 3* months	−1.269 (−6.98 to 4.442); p=NS	−1.308 (−6.714 to 4.098); p=NS	NS
*Baseline to 6* months	−2.445 (−8.453 to 3.564); p=NS	−1.285 (−6.996 to 4.426); p=NS	NS
*Baseline to 12* months	−1.56 (−7.809 to 4.689); p=NS	−2.264 (−8.225 to 3.697); p=NS	NS
*PAM score=25*			
*Baseline to 3* months	4.51 (−10.959 to 1.938); p=NS	−4.549 (−10.672 to 1.573); p=NS	NS
*Baseline to 6* months	−5.686 (−12.281 to 0.91); p=NS	−4.526 (−10.897 to 1.846); p=NS	NS
*Baseline to 12* months	−4.801 (−11.642 to 2.039); p=NS	−5.505 (−12.071 to 1.061); p=NS	NS
*PAM score=50*			
*Baseline to 3* months	−7.751 (−15.827 to 0.324); p=NS	−7.790 (−15.563 to −0.018); p=0.05	NS
*Baseline to 6* months	−8.927 (−17.024 to −0.829); p=0.031	−7.767 (−15.72 to 0.186); p=NS	NS
*Baseline to 12* months	−8.042 (−16.361 to 0.276); p=NS	−8.746 (−16.83 to −0.662); p=0.034	NS
*PAM score=75*			
*Baseline to 3* months	−10.993 (−21.168 to −0.817); p=0.034	−11.032 (−20.934 to −1.13); p=0.029	NS
*Baseline to 6* months	−12.168 (−22.284 to −2.052); p=0.018	−11.008 (−21.038 to −0.979); p=0.032	NS
*Baseline to 12* months	−11.284 (−21.594 to −0.973); p=0.032	−11.987 (−22.101 to −1.873); p=0.020	NS
*PAM score=100*			
*Baseline to 3* months	−14.234 (−26.747 to −1.721); p=0.026	−14.273 (−26.536 to −2.01); p=0.023	NS
*Baseline to 6* Months	−15.409 (−27.811 to −3.007); p=0.015	−14.249 (−26.604 to −1.895); p=0.024	NS

LUP-QOL BP, Lupus Quality of Life Questionnaire; PAM, Patient Activation Measure.

## Discussion

This study provides several key findings. While an intervention effect was not significantly associated with the reduction of bodily pain among participants, a reduction in pain was observed among both the intervention group and social support control groups for PAM scores of at least 50 units. This suggests a relationship between PAM and bodily pain, specifically that those with higher activation (self-confidence in managing symptoms) have reduced bodily pain. This finding is in alignment with prior work and emphasises the necessity for patients to actively engage in their healthcare journey to improve outcomes. Collectively, these findings show that patients who are more involved in their care—who understand their symptoms, have strategies to manage them and maintain a sense of self-efficacy—are more likely to achieve reduced pain and improved overall health. This suggests that focusing on strategies which increase engagement in care can have tangible health benefits.^39^

This study had relatively high PAM scores (average scores of 76 for both groups) based on previous work that suggested a score of at least 67 on this measure is considered ‘high patient activation’.^40^ This underscores how modifying patient activation is an important precursor of reduced pain, but future work is needed to confirm this finding.

The novelty of this study lies in part with the intervention strategy combined with the RCT research design. To our knowledge, PALS is the only peer-mentoring or social support intervention for SLE using an RCT design. Within the domain of non-pharmacological interventions in SLE, RCT designs are lacking and even fewer of these include pain as an outcome of focus. In a review of non-pharmacologic interventions RCTs in SLE, only three of the included 15 studies included pain as outcome.^41^ Among the RCTs with behavioural modification components, there were mixed results with regard to impact of pain. Greco *et al* describe a cognitive behavioural intervention and found that reduction in pain was associated with experimental condition.^42^ Brown *et al* used similar intervention strategies with education and education combined with cognitive behavioural therapy conditions but found no statistically significant differences between groups in pain reduction.^43^ This study adds to the currently mixed evidence base of RCTs examining pain. Future work is needed to parse out intervention strategies that work best for pain while using strong experimental designs. Recent work suggests that pain is a reasonable outcome target for these types of interventions. For instance, pilot findings from Allen *et al,* which describe self-management skills training through online tool, showed promising reductions in pain.^44^ Similarly, Hamad *et al* describe a family-delivered intervention including educational and cognitive components that also showed a reduction in pain among participants (quasi-experimental design). These studies suggest that further foundational work is needed to understand the best intervention methods for pain specifically in SLE.^45^ For example, future work may consider testing the most optimal suite of intervention strategies since current research suggests psychoeducational interventions and self-management strategies are helpful for reducing pain.^46^

The finding of an association between patient activation and lower bodily pain among African American women with SLE is important as it has implications for future interventions. The limited existing intervention research on this topic suggests the importance of providing social support and social relationships generally and the relationship to pain.^25 27^ However, the association between pain and social relationships is a vastly underexplored area in SLE. In a recent systematic review of interventions in SLE, only 1% of included studies focused on ‘social relations’ as an intervention strategy.^47^ Importantly, the large body of evidence exploring the patient experiences suggests that pain is a core aspect of the patient experience with SLE. These findings suggest that further work is needed to understand the best ways to modify pain. Additionally, the nebulous nature of pain may require novel consideration on management techniques to understand what methods patients are using to manage pain in SLE (eg, cannabis, complementary medicine).^48^

In future work, closer measurement of pain should be considered using recent frameworks such as nociplastic pain.^49^ The widespread prevalence of pain in SLE and general prerequisite of formative measurement work for causal arguments in experimental research designs. Additionally, using the framework of nociplastic, neuropathic and nociceptive pain categories will be helpful for increased precision in determining which intervention strategies modify which category of pain. The pain measurement used in this study, while specific to SLE, may partially explain the null intervention findings, specifically that pain was not able to be further typologised into potential tissue or organ damage (primarily nociceptive) and non-specific bodily pain (primarily nociplastic pain). Additionally, future work should consider additional groups beyond our study design (peer-mentoring condition, social support control). Since participants in our control group still got together in a support group setting, it may be that the important pieces of social support in this setting are the act of getting together and forming connections rather than specific peer-mentoring elements, which may explain our null intervention effect finding. Addition of a third experimental group without the support group component would help to clarify this.

This novel study in its centring of the health of black women. The disproportionate burden of SLE on black women, and racially minoritised women generally, suggests that interventions need to attend to the sociocultural specificities of living as a black or racially minoritised woman.^11^ This study attends to this by relying on important formative work, which focused on specific contexts of SLE management. This was a strength of the intervention and fits into recent reviews, which suggest that interventions which are more responsive to cultural specifics to be more effective and may reduce inequities.^50^

There are several limitations of the present study. In the intervention delivery, participants and study team members knew the treatment conditions following randomisation, which could have introduced a source of response bias impacting internal validity of the study. An additional limitation is the risk of experimental condition diffusion given that participants were recruited from the same general geographic area. Additionally, there was inconsistent collection of the additional time spent with peers, so we were unable to detect a dosage effect. Specifically, it was observed that some participants engaged more with peers and mentors beyond the study protocol, and these interactions were not measured. Finally, the initial power analysis pertained to change in the overall LUP-QOL measure. The present analysis focused on the pain subscale of LUP-QOL, so it is possible that the present analysis was underpowered.

The main findings from this study comparing peer-mentor mediated, tailored chronic disease self-management with a social support control condition suggested that high levels of patient activation were associated with reduced pain across both groups. While an experimental effect was not observed, additional work is needed to further typologise the varieties of pain experienced by people living with SLE, in addition to understanding the role of social relations and support. Specifically, additional mechanistic work is needed to understand the role of patient activation and to identify potential mediators and moderators of peer-mentoring and pain among people living with SLE and chronic disease populations more broadly. Findings from this future research will influence intervention development by suggesting the most potentially efficacious strategies for intervention testing. In conjunction with an approach that centres patient experiences in the design and testing of interventions, we have the potential to meaningfully impact pain and quality of life, several of the most persistent unmet needs among people living with SLE.

## Supplementary material

10.1136/lupus-2025-001712 online supplemental file 1

## Data Availability

Data are available upon reasonable request.

## References

[1] Falasinnu T, Drenkard C, Bao G (2021). The Problem of Pain in Systemic Lupus Erythematosus: An Explication of the Role of Biopsychosocial Mechanisms. J Rheumatol.

[2] Moses N, Wiggers J, Nicholas C (2005). Prevalence and correlates of perceived unmet needs of people with systemic lupus erythematosus. Patient Educ Couns.

[3] Jiang TE, Pascual AP, Le N (2024). The Problem of Pain in Lupus: Epidemiological Profiles of Patients Attending Multidisciplinary Pain Clinics. Pain Manag Nurs.

[4] Lee J, Lin J, Suter LG (2019). Persistently Frequent Emergency Department Utilization Among Persons With Systemic Lupus Erythematosus. *Arthritis Care & Research*.

[5] Stockl A (2007). Complex syndromes, ambivalent diagnosis, and existential uncertainty: the case of Systemic Lupus Erythematosus (SLE). Soc Sci Med.

[6] Somers EC, Marder W, Cagnoli P (2014). Population-based incidence and prevalence of systemic lupus erythematosus: the Michigan Lupus Epidemiology and Surveillance program. *Arthritis Rheumatol*.

[7] Dall’Era M, Cisternas MG, Snipes K (2017). The Incidence and Prevalence of Systemic Lupus Erythematosus in San Francisco County, California: The California Lupus Surveillance Project. *Arthritis Rheumatol*.

[8] Lim SS, Bayakly AR, Helmick CG (2014). The incidence and prevalence of systemic lupus erythematosus, 2002-2004: The Georgia Lupus Registry. *Arthritis Rheumatol*.

[9] Izmirly PM, Wan I, Sahl S (2017). The Incidence and Prevalence of Systemic Lupus Erythematosus in New York County (Manhattan), New York: The Manhattan Lupus Surveillance Program. Arthritis Rheumatol.

[10] Garg S, Bartels CM, Bao G (2023). Timing and Predictors of Incident Cardiovascular Disease in Systemic Lupus Erythematosus: Risk Occurs Early and Highlights Racial Disparities. J Rheumatol.

[11] Lim SS, Drenkard C (2020). Health disparities in rheumatic diseases: Part I, An Issue of Rheumatic Disease Clinics of North America, E-Book: Health disparities in rheumatic diseases.

[12] Demas KL, Costenbader KH (2009). Disparities in lupus care and outcomes. Curr Opin Rheumatol.

[13] Murphy AE, Minhas D, Clauw DJ (2023). Identifying and Managing Nociplastic Pain in Individuals With Rheumatic Diseases: A Narrative Review. *Arthritis Care & Research*.

[14] Kosek E, Cohen M, Baron R (2016). Do we need a third mechanistic descriptor for chronic pain states?. Pain.

[15] Rogers JL, Eudy AM, Pisetsky D (2021). Using Clinical Characteristics and Patient‐Reported Outcome Measures to Categorize Systemic Lupus Erythematosus Subtypes. *Arthritis Care & Research*.

[16] Eudy AM, Clowse ME, Corneli A (2024). The Type 1 & 2 systemic lupus erythematosus model: Perspectives of people living with systemic lupus erythematosus. Lupus (Los Angel).

[17] Pisetsky DS, Eudy AM, Clowse MEB (2021). The Categorization of Pain in Systemic Lupus Erythematosus. Rheum Dis Clin North Am.

[18] Leung J, Baker EA, Kim AHJ (2021). Exploring intentional medication non-adherence in patients with systemic lupus erythematosus: the role of physician-patient interactions. Rheumatol Adv Pract.

[19] Wagner EH (1998). Chronic disease management: what will it take to improve care for chronic illness?. Eff Clin Pract.

[20] Thomas RE, Lorenzetti DL, Spragins W (2013). Systematic review of mentoring to prevent or reduce tobacco use by adolescents. Acad Pediatr.

[21] Heisler M (2010). Different models to mobilize peer support to improve diabetes self-management and clinical outcomes: evidence, logistics, evaluation considerations and needs for future research. Fam Pract.

[22] Danoff-Burg S, Friedberg F (2009). Unmet needs of patients with systemic lupus erythematosus. Behav Med.

[23] Shi Y, Li M, Liu L (2021). Relationship between disease activity, organ damage and health-related quality of life in patients with systemic lupus erythematosus: A systemic review and meta-analysis. Autoimmun Rev.

[24] Jordan J, Thompson NJ, Dunlop-Thomas C (2019). Relationships among organ damage, social support, and depression in African American women with systemic lupus erythematosus. Lupus (Los Angel).

[25] Fischin J, Chehab G, Richter JG (2015). Factors associated with pain coping and catastrophising in patients with systemic lupus erythematosus: a cross-sectional study of the LuLa-cohort. *Lupus Sci Med*.

[26] Bae S-C, Hashimoto H, Karlson EW (2001). Variable effects of social support by race, economic status, and disease activity in systemic lupus erythematosus. J Rheumatol.

[27] Holtzman S, Newth S, Delongis A (2004). The role of social support in coping with daily pain among patients with rheumatoid arthritis. J Health Psychol.

[28] Lorig K, Ritter PL, Plant K (2005). A disease‐specific self‐help program compared with a generalized chronic disease self‐help program for arthritis patients. *Arthritis & Rheumatism*.

[29] Lorig K, Laurin J, Gines GE (1984). Arthritis self-management. A five-year history of a patient education program. Nurs Clin North Am.

[30] (1987). The systemic lupus erythematosus self-help course: program guidelines and procedures manual.

[31] Flournoy-Floyd M, Ortiz K, Egede L (2018). “We Would Still Find Things to Talk About”: Assessment of Mentor Perspectives in a Systemic Lupus Erythematosus Intervention to Improve Disease Self-Management, Empowering SLE Patients. J Natl Med Assoc.

[32] Feldman CH, Bermas BL, Zibit M (2013). Designing an intervention for women with systemic lupus erythematosus from medically underserved areas to improve care: a qualitative study. Lupus (Los Angel).

[33] Williams EM, Egede L, Oates JC (2019). Peer approaches to self-management (PALS): comparing a peer mentoring approach for disease self-management in African American women with lupus with a social support control: study protocol for a randomized controlled trial. Trials.

[34] Peterson MGE, Horton R, Engelhard E (1993). Effect of counselor training on skills development and psychosocial status of volunteers with systemic lupus erythematosus. *Arthritis & Rheumatism*.

[35] Kang M, Ragan BG, Park J-H (2008). Issues in outcomes research: an overview of randomization techniques for clinical trials. J Athl Train.

[36] Webster K, Cella D, Yost K (2003). The Functional Assessment of Chronic Illness Therapy (FACIT) Measurement System: properties, applications, and interpretation. Health Qual Life Outcomes.

[37] Shikiar R, Howard K, Yu E (2006). Validation of LUP-QOL: a lupus-specific measure of health-related quality of life (hrql).

[38] Hibbard JH, Stockard J, Mahoney ER (2004). Development of the Patient Activation Measure (PAM): conceptualizing and measuring activation in patients and consumers. Health Serv Res.

[39] Wolfe F, Michaud K (2009). Predicting depression in rheumatoid arthritis: The signal importance of pain extent and fatigue, and comorbidity. *Arthritis & Rheumatism*.

[40] Wang Z, Song Y, Ou L (2024). Factors affecting patient activation among patients with systemic lupus erythematosus. Sci Rep.

[41] Fangtham M, Kasturi S, Bannuru RR (2019). Non-pharmacologic therapies for systemic lupus erythematosus. Lupus (Los Angel).

[42] Greco CM, Rudy TE, Manzi S (2004). Effects of a stress-reduction program on psychological function, pain, and physical function of systemic lupus erythematosus patients: a randomized controlled trial. Arthritis Rheum.

[43] Brown RT, Shaftman SR, Tilley BC (2012). The Health Education for Lupus Study: A Randomized Controlled Cognitive-Behavioral Intervention Targeting Psychosocial Adjustment and Quality of Life in Adolescent Females With Systemic Lupus Erythematosus. Am J Med Sci.

[44] Allen KD, Beauchamp T, Rini C (2021). Pilot study of an internet-based pain coping skills training program for patients with systemic Lupus Erythematosus. BMC Rheumatol.

[45] Hamad AH, Ragab II, Zytoon HK (2024). Effect of Non-Pharmacological Nursing Interventions on Fatigue, Pain and Quality of Life for Patients with Systemic Lupus Erythematosus. *Zagazig Nursing Journal*.

[46] Poole JL, Bradford JD, Siegel P (2019). Effectiveness of Occupational Therapy Interventions for Adults With Systemic Lupus Erythematosus: A Systematic Review. Am J Occup Ther.

[47] Tsoi A, Gomez A, Boström C (2024). Efficacy of lifestyle interventions in the management of systemic lupus erythematosus: a systematic review of the literature. Rheumatol Int.

[48] Habib G, Khazin F, Artul S (2021). The Effect of Medical Cannabis on Pain Level and Quality of Sleep among Rheumatology Clinic Outpatients. Pain Res Manag.

[49] Marchand S (2024). The Pain Phenomenon.

[50] Leung J, Sekar S, Madrigal L (2025). A Scoping Study of Cultural Adaptation Frameworks. Health Promot Pract.

