# Supplementary material for: Does social support reduce bodily pain among African American women with SLE? Findings from a randomised controlled trial
Source: Lupus Sci Med. 2025 Oct 20;12(2):e001712. doi: 10.1136/lupus-2025-001712 (PMC12542739; doi:10.1136/lupus-2025-001712)
Supplement: online supplemental file 1 [file lupus-12-2-s001.pdf]

Williams et al. Trials (2019) 20:529
https://doi.org/10.1186/s13063-019-3580-4

Trials

STUDY PROTOCOL Open Access

Peer approaches to self-management (PALS): comparing a peer mentoring approach for disease self-management in African American women with lupus with a social support control: study protocol for a randomized controlled trial

Edith M. Williams[1*], Leonard Egede[2], Jim C. Oates[3,4], Clara L. Dismuke[5], Viswanathan Ramakrishnan[1], Trevor D. Faith[3], Hetlena Johnson[6] and Jillian Rose[7]

Abstract

Background: Systemic lupus erythematosus (SLE or lupus) is a chronic autoimmune disease that is associated with increased morbidity, mortality, healthcare costs and decreased quality of life. African Americans in the USA have three to four times greater prevalence of SLE, risk of developing SLE at an earlier age, and SLE-related disease activity, damage, and mortality compared with Caucasians, with the highest rates experienced by African American women. There is strong evidence that patient-level factors are associated with outcomes, which justifies targeting them with intervention. While evidence-based self-management interventions that incorporate both social support and health education have reduced pain, improved function, and delayed disability among patients with SLE, African Americans and women are still disproportionately impacted by SLE. Peer mentoring interventions are effective in other chronic conditions that disproportionately affect minorities, such as diabetes mellitus, HIV, and kidney disease, but there is currently no empirically tested peer mentoring intervention developed for patients with SLE. Preliminary data from our group suggest that peer mentoring improves self-management, reduces disease activity, and improves health-related quality of life (HRQOL) in African American women with SLE.

Methods: This study will test an innovative, manualized peer mentorship program designed to provide modeling and reinforcement by peers (mentors) to other African American women with SLE (mentees) to encourage them to engage in activities that promote disease self-management. Through a randomized, "mentored" or "support group" controlled design, we will assess the efficacy and mechanism(s) of this intervention in self-management, disease activity, and HRQOL.

(Continued on next page)

* Correspondence: wiled@musc.edu
[1]Department of Public Health Sciences, Medical University of South Carolina, 135 Cannon Street, Suite CS303D, Charleston, SC 29425, USA
Full list of author information is available at the end of the article

© The Author(s). 2019 **Open Access** This article is distributed under the terms of the Creative Commons Attribution 4.0 International License (http://creativecommons.org/licenses/by/4.0/), which permits unrestricted use, distribution, and reproduction in any medium, provided you give appropriate credit to the original author(s) and the source, provide a link to the Creative Commons license, and indicate if changes were made. The Creative Commons Public Domain Dedication waiver (http://creativecommons.org/publicdomain/zero/1.0/) applies to the data made available in this article, unless otherwise stated.

(Continued from previous page)

Discussion: This is the first study to test peer mentorship as an alternative strategy to improve outcomes in African American women with SLE. This could result in a model for other programs that aim to improve disease self-management, disease activity, and HRQOL in African American women suffering from chronic illness. The peer mentoring approach is uniquely fitted to African Americans, and this intervention has the potential to lead to health improvements for African American women with SLE that have not been attainable with other interventions. This would significantly reduce disparities and have considerable public health impact.

Trial registration: ClinicalTrials.gov, NCT03734055. Registered on 27 November 2018.

Keywords: Systemic lupus erythematosus, African American, Women, Peer mentoring, Behavioral intervention, Self-management

Background

SLE (or lupus) is a chronic autoimmune disease affecting over 250,000 individuals, which is marked by acute periodic flare ups of symptoms impacting any organ system and resulting in potentially life-threatening complications [1–3]. Health-related quality of life (HRQOL) of patients with SLE is also significantly worse and affects all health domains at an earlier age compared to patients with other common chronic diseases and to women in the general US population [4–10]. In the USA, the highest lupus morbidity and mortality rates are among African American women [2, 11, 12]. SLE affects approximately 1 in 250 African American women of childbearing age, and African Americans overall have three to four times greater prevalence of lupus, risk of developing lupus at an earlier age, and lupus-related disease activity, damage, and mortality compared with Caucasians [13–17].

Evidence-based self-management interventions, designed to enhance social support and provide health education among patients with lupus, have reduced pain, improved function, and delayed disability [12, 18–24], but African Americans and women are still disproportionately impacted by SLE [13, 14, 25–28]. Persistent disparities may be due to the non-responsiveness of existing programs to the unique needs of African Americans and/or women with SLE [12, 18, 29–35]. Previous results have shown that African American patients with SLE were more likely than white patients to have higher levels of unmet needs related to health services and information [29, 31, 36]. These domains have included issues such as (1) understanding the medical regimen, including considerations around depression, medication concerns (possible side effects and interactions), and physical symptoms (pain and fatigue); (2) trust in the provider; (3) communication with providers; (4) receiving adequate information from medical staff about treatment side effects; (5) having access to telephone support and advisory services; and (6) having assistance with knowing which symptoms should trigger a doctor visit [29, 31, 37, 38].

Peer mentors are usually individuals who have successfully coped with a similar condition as their mentees [39]. In formal interventions, mentors receive training focused on communication skills, including empathetic listening, helping mentees clarify life goals, and problem solving with the aim of having the mentor support the mentee [40]. In studies of predominantly low income and minority populations peer mentors have been shown to help support healthy behaviors including breast feeding, smoking cessation, increased physical activity, and maintenance of weight loss [41–48], along with improved medication adherence and blood glucose monitoring in trials of people with diabetes mellitus [49–55]. In the Peer approaches to lupus self-management (PALS) intervention pilot study, mentees showed a trend toward lower disease activity, higher quality of life, lower pain symptoms and higher social support (effect sizes > 0.3) following participation in the intervention. In addition, both mentees and mentors gave very high scores for perceived treatment credibility and service delivery [56, 57].

Using a randomized controlled design, this study will test a peer mentoring intervention for African American women with SLE, wherein modeling and reinforcement of disease self-management skills by peers (mentors) to other African American women with SLE (mentees) will be achieved through a combination of educational and informal phone or video interactions with each other, along with the use of validated measures of patient-reported outcomes and clinical indicators of disease activity to assess the efficacy of the program.

A primary aim of the program will be to determine the efficacy of a peer mentorship intervention in African American women with SLE on disease self-management and HRQOL, with the hypothesis that mentees will report improved disease self-management and HRQOL, as measured by the Patient Activation Measure (PAM) and Lupus Quality of Life Questionnaire (LUP-QOL), compared with the social support control group, at 12 months post-randomization. A secondary aim will be to determine the

cost and cost-effectiveness of a peer mentorship intervention on disease self-management, disease activity, and HRQOL, in African American women with SLE, with the hypothesis that a peer mentorship intervention in African American women with SLE will be cost effective at improving disease self-management, disease activity, and HRQOL, as measured by quality-adjusted life years (QALYS), compared with the social support control group.

Methods/design

Study overview

The PALS study is a randomized controlled trial designed to examine whether a new, culturally tailored peer mentoring intervention improves disease self-management, indicators of disease activity, and HRQOL in African American women with SLE. African American women with active SLE will be recruited as mentees and peer mentors. Our Standard protocol items: recommendation for interventional trials (SPIRIT) checklist (Additional file 1) and figure (Fig. 1) identify the procedures and assessments to be carried out as part of the PALS study and outline when procedures/assessments occur throughout the study. We will recruit 300 mentees (150 mentored and 150 support group) and up to 60 mentors. Figure 2 shows that as part of each wave, mentors (n = 20) will be trained to deliver intervention content prior to being paired with up to three mentees (n = 50). The peer mentoring intervention will comprise twelve 60-min telephone or video sessions carried out across the course of 24 weeks. In each wave, social support controls (n = 50) will participate in a lupus support group created for this project, on the same schedule as peer mentoring sessions. All participants (mentees, mentors, and social support controls) will be assessed using validated measures of patient-reported outcomes and clinical indicators of disease activity at baseline, mid-intervention (3 months from baseline), immediately post-intervention (6 months from baseline), and 6 months post-intervention (12 months from baseline). For each wave, outcomes for mentees randomized to the mentored group will be compared with the outcomes of mentees randomized to the support group. A booster session will be incorporated for all participants (mentored and support group) at 3 months post-intervention to encourage retention [58].

Study population

The study population are individuals with SLE at Medical University of South Carolina (MUSC) clinics. All patients have American College of Rheumatology (ACR) criteria and disease activity information available, as well as quality of life measures available in the database. All patients with SLE meet at least four components of the 1997 ACR revised criteria for SLE [59]. Inclusion criteria for mentees and mentors include (1) African American race/ethnicity and female gender; (2) clinical diagnosis of SLE from a physician, according to ACR revised criteria for SLE [59]; and (3) 18 years of age or older. Additional inclusion criteria for mentors include (1) disease duration > 2 years; (2) able to attend scheduled training sessions; and (3) willing to provide one-on-one support to up to three African American women with SLE. Mentees who participated in the pilot study and other related behavioral trials will be ineligible to participate in this study as a mentee, but could participate as a mentor if they meet other eligibility criteria.

Recruitment

Mentees (n = 300; 150 mentored, 150 support group) will be recruited by a direct mailing to female, African American patients with lupus currently enrolled in the MUSC P30 Core Center for Clinical Research (CCCR)

Month	Screening/ Recruitment	Surveys administered	Physician evaluation of disease activity	Mentor Training	Bi-weekly mentoring sessions	Cost effectiveness evaluation	Participants w/ adverse events withdrawn	Booster Session
1	X	X	X					
2	X	X	X					
3	X	X	X					
4	X	X	X					
5	X	X	X					
6	X	X	X	X				
7					X			
8					X			
9		X	X		X	X	X	
10					X			
11					X			
12		X	X		X	X		
13								
14								
15								X
16								
17								
18		X	X			X		

Fig. 1 SPIRIT figure of participant activity within one cohort

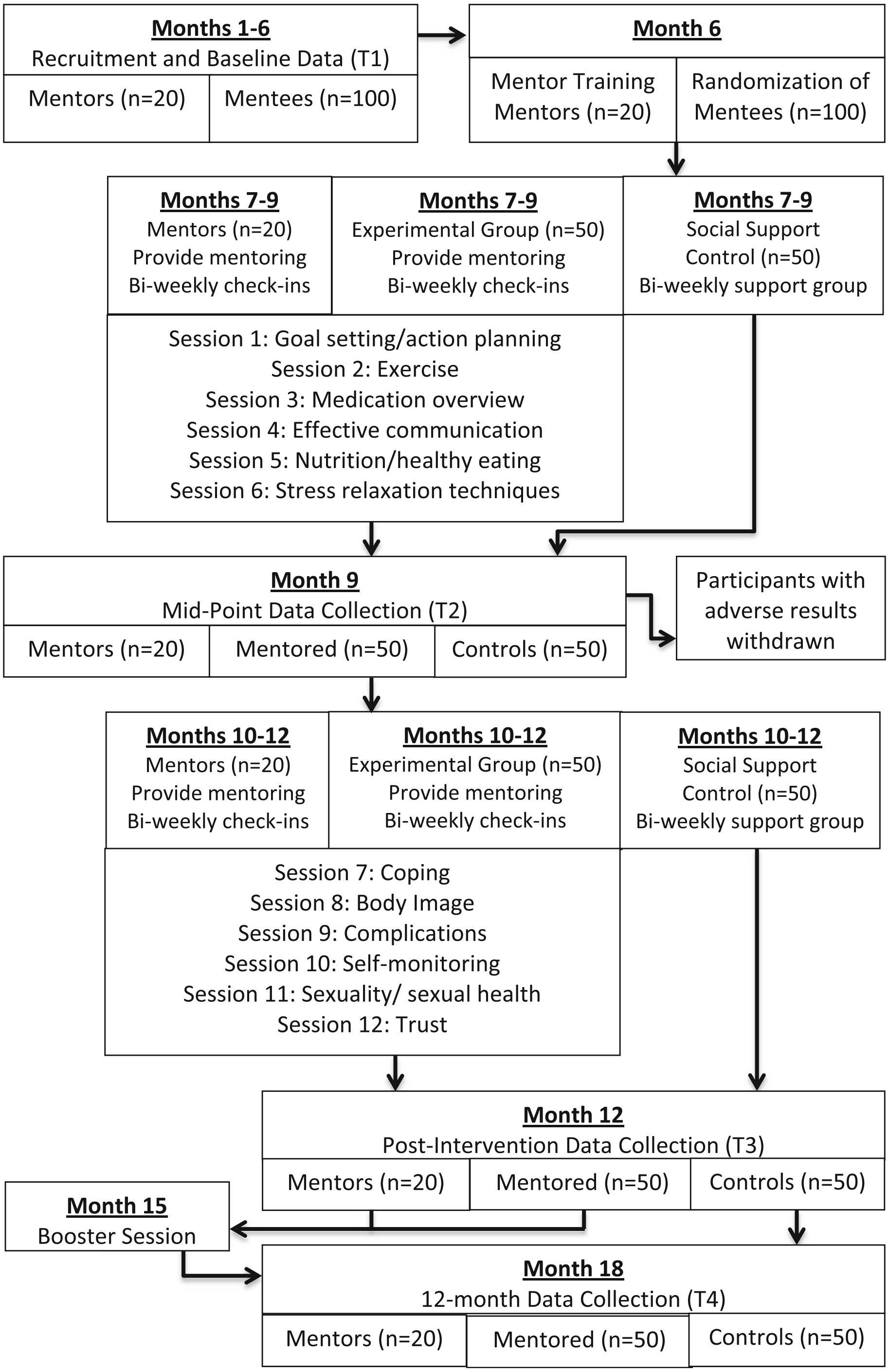

Fig. 2 Procedural flow chart for mentors, mentored participants, and controls

SLE database who have agreed to future contact and lupus patients from the MUSC clinics who are not in the registry. Flyers containing the same information as in the recruitment letters will be posted in MUSC lupus clinics and shared with other stakeholders.

Potential peer mentors will first be invited from among the PALS pilot study participants (mentees and mentors) ($n = 30$) [56]. Potential peer mentors who are considered competent in the management of their conditions will be identified by MUSC rheumatologists as needed (up to 60), and subsequently trained by the Principal Investigator (PI). We will mail out recruitment letters that will explain the study and provide participants a number to call if they are interested in participating. If

eligibility criteria are met, the screening/enrollment visit will be scheduled. Psychosocial status will be assessed as part of the mentor screening interview with the PI, using the psychological scales of the Arthritis Impact Measurement Scales (AIMS), Arthritis Helplessness Index (AHI), Wallston General Perceived Competence Scale, University of California at Los Angeles (UCLA) Loneliness Scale, Rosenberg Self-Esteem, Campbell Personal Competence Index, Carkhuff Communication and Discrimination Skills Inventories, and the Applied Knowledge Assessment (AKA) scale [60]. The PI will make a determination of competence, maturity, emotional stability, and verbal communication skills after overall assessment during the screening interview and training.

Recruitment will occur in three waves. Within each wave, each mentor will be assigned all of their mentees at one time to ensure that intervention activities occur within the same 12-month period. As mentor:mentee quads (one mentor, three mentees) are identified, they will attend an introductory session together, during which the mentoring process will be discussed, including time commitment, roles, responsibilities, benefits, and ground rules, and mentees and peer mentors will have the opportunity to ask questions and make informed decisions about their ability to fully participate in the intervention.

Mentor training

The principal roles of the peer mentors are to (1) provide information about SLE, SLE-related behaviors, thoughts, and feelings, and the nature of recommended treatments; (2) provide social support to alleviate the mentee's sense of social isolation; (3) enhance and reinforce the mentee's sense of self-efficacy to manage their condition; and (4) encourage the mentee to participate actively in the recommended self-management skills building therapy. Mentors will be trained in conversational strategies to help them meet the objectives without being overly directive and will be instructed not to give clinical advice [61]. Upon enrollment, peer mentors will receive 12 h of training, broken into two 6-h blocks, prior to working with mentees [61]. Mentors will be given a written manual presenting all the material in detail for their ongoing reference. The training manual was developed in collaboration with social work leadership from Hospital for Special Surgery's LupusLine® Program. The program, led by the Department of Social Work Programs, is a free telephone counseling service staffed by trained volunteers who have SLE or are close family or friends of people living with lupus [60, 62].

Mentee pairing

After enrollment and completion of baseline assessments, mentees will be matched with peer mentors based on as many specific shared concerns of their experiences as possible. Potential matching areas include disease symptoms, parenting, work-related concerns, similarity of life stage (including age) [63] and demographics (including area of residence), similarity of personality characteristics, and peer mentor availability, and will be assessed as part of the screening process.

Randomization

Mentees recruited for the experimental (mentored) or control (support group) portion of the study will be randomized using a block randomization procedure to assure equal sample sizes in the mentored and control groups. Using a block size of three, participants will be assigned to the appropriate treatment condition as they enroll in the study until the block is completed. Then the following three participants will be assigned based on the next block [64]. Once a participant is randomized and attends the first session, she will be entered into the study and included in the intent-to-treat analysis plan. Participants will remain blinded to group allocation until after the completion of baseline assessment.

Peer mentoring intervention

Trained mentors will deliver the intervention program to mentees randomized to the experimental (mentored) condition. The program will consist of 12 sessions of peer mentoring that will include one standard educational session by telephone or video for approximately 60 min every 2 weeks. Biweekly content has been adapted from the six modules of the Chronic Disease Self-Management Program (CDMP), Arthritis Self-Management Program (ASMP), and Systemic Lupus Erythematosus Self-Help (SLESH) course [19, 65], and further tailored to African American women with six added sessions based on cultural issues reported as important to African Americans in earlier research conducted by the PI [66, 67] and documented unmet needs in African American patients with SLE [68, 69].

Tailoring of the PALS intervention

To address unmet needs around understanding the medical regimen, including considerations around depression, medication concerns, and physical symptoms, culturally relevant sessions on "Complications" and "Self-monitoring" were developed. In response to unmet needs around trust in the provider, communication with providers, and receiving adequate information from medical staff about treatment side effects, sessions on "Coping" and "Trust" were developed. Last, unmet needs around having access to telephone support and advisory services and having assistance with knowing which symptoms should trigger a doctor visit [29, 31, 36] are addressed by the PALS study design (i.e., telephone/

video delivery of intervention) and sessions devoted to less frequently discussed topics of "Body image" and "Sexuality/sexual health". The PALS pilot was used for initial refinement of the intervention protocol. We analyzed qualitative responses that were collected as part of weekly mentee check-ins, mentor logs, and the end-of study focus group. Themes that emerged included (1) interpersonal, familial and romantic relationships; (2) individual experiences of living with SLE; and (3) physician-patient relationships. Additional themes emphasized how the intervention worked bi-directionally wherein both mentors and mentees were empowered toward greater disease self-efficacy. We found that (1) empowerment was facilitated/achieved by mentors taking their mentorship responsibilities seriously and seeking several avenues for collaboratively developing success with their mentees; (2) mentors felt empowered through being able to discuss topics that they felt were often marginalized by healthcare professionals, such as sexuality; and (3) the intervention encouraged reciprocity. Such dynamic discussions served as a participative approach to determining which components of the intervention were most useful to participants. Based on observed themes, unique concerns of our study population have been built into the proposed intervention. Specific themes have been incorporated into educational sessions and the PALS implementation plan and training protocols, to ensure that culture-bound myths and concerns about SLE are addressed in this cultural group [70].

Control intervention (support group)

Mentees randomized to the social support control group will be enrolled in a lupus support group designed specifically for this project. Unlike traditional support-group meeting formats - that are open to all patients with lupus, family members, friends and supporters, are advertised publicly, are implemented by a trained facilitator; and generally include a specific discussion topic or an informative presentation - the PALS-specific support group will be limited to PALS control participants, be moderated by a PALS study coordinator who will not provide any information or discussion topics, and will simply provide a meeting session for social support control participants to interact on a bi-weekly basis.

Treatment fidelity

At the onset of the study, peer mentors will receive extensive training [71, 72]. In addition, mentors will receive ongoing oversight of peer mentoring sessions. Training will consist of two full days of information and role-playing and then one-day booster sessions in years 2–5 to minimize drift in peer mentoring skills [58]. After initial training, peer mentors will continue to meet with the PI bi-weekly to identify challenges and reinforce the guidelines for peer mentors [61]. Mentors will be required to submit logs of the number of calls made, number of hours spent with mentees, and content covered during that two-week period, in order to be compensated. Mentees will be surveyed every 2 weeks to assess the frequency and duration of calls, other interactions with their mentor, and whether specific content has been covered. Additionally, a subset of sessions will be recorded to allow direct evaluation of the contents of interactions.

Data collection

Mentees will be assessed at baseline, mid-intervention (3 months from baseline), immediately post-intervention (6 months from baseline), and 6 months post-intervention (12 months from baseline). Physical examination and laboratory evaluation will be achieved by in-person clinic visit when recent Systemic Lupus Disease Activity Index (SLEDAI) scores are not available in the database record of a given participant. Social support control participants will complete assessments on the same schedule as mentored participants. Given evidence that peer support may be just as beneficial to the supporters as it is to the person being supported [73, 74], mentors will be assessed on the same schedule as mentored and control participants, using the same tools.

Primary outcome measures

Quality of life will be assessed using the LUP-QOL. The LUP-QOL incorporates the Medical Outcomes Study (MOS) Short Form 36 Health Survey (SF-36) and the Functional Assessment of Chronic Illness Therapy-Fatigue (FACIT-F), which are reliable and valid instruments that are frequently used in quality of life studies of persons with lupus [75, 76].

Self-management will be measured by the PAM [77, 78], which assesses an individual's knowledge, skill, and confidence for managing their health and healthcare. Individuals who measure high on this assessment typically understand the importance of taking a proactive role in managing their health and have the skills and confidence to do so.

Secondary outcome measures

Treatment credibility will be assessed as differences in outcome expectancy using a modified treatment credibility scale developed by Borkovec and Nau (1972). Four of the questions will be used for this study, with 10-point Likert scales. These include questions on how logical the treatment seems, how confident participants are about treatment, and their expectancy of success.

Satisfaction with care will be measured with a previously validated general scale to measure satisfaction/

dissatisfaction with health care. The 2-item scale ranges from 1 (strongly agree) to 5 (strongly disagree).

Healthcare utilization will be assessed using Stanford Patient Education Research Center Questionnaires [79, 80] assessing medical outcomes such as hospital visits. Questionnaires have been adapted to include questions related to use of other services, such as emergency department visits, other medical care resources of importance to patients, economic and financial barriers to use of care outside the hospital setting including loss of time at work/productivity, and any issues related to recidivism of patients once they no longer have mentor support [81–83].

Predictor variables

Predictors that might distinguish participants who benefit from the interventions include demographic factors, pre-existing disease damage, coping, depression, anxiety, perceived stress, and health literacy. Measures of social support, trust, and patient-centered care will be administered to test whether unmet needs around trust in the provider, communication with providers, receiving adequate information from medical staff about treatment side effects, and having access to telephone support and advisory services are better met in the group receiving the peer mentorship intervention compared to the group receiving social support.

Demographics: previously validated items from the 2002 National Health Interview Survey (NCHS 2004) will be used to capture age, marital status, education, household income, and health insurance. The 28-item Brief Index of Lupus Damage (BILD) was developed as a patient-reported measurement of lupus disease damage designed to quantify cumulative organ damage due to SLE regardless of attribution. The self-administered version of the BILD has been validated in a predominantly African American independent community-based cohort of patients with SLE from the Southeastern USA [25].

Coping: coping will be assessed by the Arthritis Self-Efficacy Scale pain and other symptoms sub-scale [84], which consists of 11 items designed to measure confidence in one's ability to manage the pain, fatigue, frustration, and other aspects of disease [22].

Depression: the Patient Health Questoinnaire (PHQ)-9 is a brief questionnaire that scores each of the 9 DSM-IV criteria for depression as 0 (not at all) to 3 (nearly every day). PHQ-9 scores > 10 or = 10 have sensitivity of 88% and specificity of 88% for classification of major depression [85].

Anxiety: general anxiety disorder (GAD) will be assessed using the 7-item General Anxiety Disorder-7 (GAD-7) scale. This is a valid and efficient tool for screening for GAD and assessing its severity in clinical practice and research [86].

Perceived stress: the Perceived Stress Scale (PSS) is a 4-item scale that assesses the degree to which the respondent finds situations stressful [87]. Responses range from 0 (never) to 4 (very often) and questions ask about the frequency of feelings related to events in the previous month. The Cronbach alpha value is 0.69 and scores are strongly correlated with stress, depression, and anxiety.

Chew Health Literacy Screening: the Chew Health Literacy Screening Survey [88] is a 3-item instrument designed to rapidly screen patients for potential health literacy problems. To test whether unique needs are better met in the group receiving the peer mentorship intervention compared to the group receiving social support, this instrument will be adapted to include questions about understanding the medical regimen, including considerations around depression, medication concerns (possible side effects and interactions), and physical symptoms (pain and fatigue), and knowing which symptoms should trigger a doctor visit [29, 31, 36].

Social support: the Medical Outcomes Study (MOS) Social Support Survey will be used to measure social support [89]. The total scale (d = 0.97) and subscales (d = 0.91–0.96) have high internal consistency, good criterion and discriminant validity, and one-year test-retest reliability (0.72–0.76).

Trust: trust will be measured using the 17-item Multidimensional Trust in Health Care Systems Scale (MTHCSS) [90]. Items are scored on a 5-point Likert scale with scores ranging from 5 (strongly agree) to 1 (strongly disagree). The higher scores represent greater trust in the healthcare systems.

Patient-centered care: patient-centered care will be measured using the Modified Picker Survey. It is a 7-item scale that measures patients' experience with the physician. Scores range from 1 (always) to 4 (never).

Disease activity

Disease activity will be assessed using both physician assessment and patient-reported outcome measure. The Systemic Lupus Activity Questionnaire (SLAQ) [91] asks a single Patient Global Assessment (PGA) question about presence and severity of lupus activity over the past month, questions on 24 specific symptoms of disease activity, and a single numerical rating scale (NRS) asking the patient to rate disease activity on a scale of 0–10 over the past 3 months. Use of immunomodulatory drugs and prednisone (total dose and tapers) will also be assessed. For physician assessment of disease activity, the SLEDAI has been individually validated and found reliable in clinical trials. SLEDAI scores are routinely collected as part of regular visits and clinical, demographic, genetic, disease activity/damage, genetic, and laboratory data are stored in the longitudinal web-based

SLE database at MUSC. SLEDAI scores for each participant will be extracted from the database when available for dates within the same month as baseline, mid-intervention, and post-intervention data collection points. When scores are not available in the database, the participant will be scheduled for a clinic visit that will include vitals, blood collection, and laboratory values to ascertain the SLEDAI score. The SLEDAI is a multicomponent, 24-question survey of clinical and laboratory signs and symptoms used as a representation of a phsyician's assesment of a patient's disease activity over the last 30 days. Items are weighted based on their severity ranging from a multiplier of 8 to no multiplier (i.e., 1). The maximum "score" for the test is 105 [92]. Validated clinically meaningful changes in SLEDAI scores are − 6 for improvements and + 8 for worsening disease activity [93].

Cost effectiveness

Resource use and cost information will be collected to inform a well-designed economic study of the cost-effectiveness of the use of peer mentors for patients with SLE in the acute care setting. Cost of the intervention will include all personnel, equipment, supply and space cost associated with training, and use of peer mentors, in real-time dollar values. MUSC inpatient and outpatient costs of healthcare utilization of any MUSC services will be collected from MUSC administrative billing data based on International Classification of Diseases (ICD)9/10 codes, Medicare Diagnosis Related Group (MSDRG), and current procedural technology (CPT) codes related to lupus to estimate distributions of cost for the medical care resources used. Resource use and cost data will be accessed through the Services, Pricing, and Application for Research System, which is available to MUSC-based investigators under the MUSC Clinical and Translational Science Award. The system allows for easy access to pricing for services across the MUSC campus and its providers and focuses on billing compliance and budgetary analysis. In order to extract data from the MUSC record systems, services are requested through an online portal and data are then provided through direct consultation. Within the SPARC system, members of the study team will also be able to track service utilization and pricing throughout the duration of the study. Questionnaire responses will be used to ascertain care resources that patients use during the study period from other hospitals or entities who are not part of the MUSC record system.

Statistical analysis

Sample size determination and power analysis The sample size calculation and power analyses are based on the primary outcome of change in HRQOL between baseline and 12 months post intervention. The minimum sample size was based on detecting a clinically meaningful difference of 0.35 standard deviation units (medium effect) based on prior studies [56, 66, 94–98]. Assuming three measurement time points, level of significance $\alpha = 0.05$, two-tailed comparison, correlation between pairs of measurements within participants (interclass correlation) no larger than $\rho = 0.6$, and compound symmetry covariance structure, we estimate that 123 participants per group (total 246) are needed to detect a standardized effect size of at least 0.35 sd with 80% power. This sample size includes 20% inflation for attrition at 12 months. This effect size is robust enough to provide sufficient power for the outcomes listed previously and is consistent with data from our pilot study of 20 mentees and 7 mentors [56]. The pre-post differences in the outcomes (such as overall social support, positive social interaction, tangible support, vitality, emotional support, social functioning, general health, coping, etc.) ranged from 0.35 to 0.88 sd units. Although these calculations account for within-patient clustering through the intraclass correlation mentioned above, clustering within mentors and mentees are assumed to have minimal intraclass correlation based on pilot data, especially since the cluster sizes would be 3 at most in a given wave. However, a multi-level model will be used in the analysis to verify this. Since the effect size planned is conservative, if the clustering leads to higher intra-class correlation, we would still be able to detect meaningful differences. We will also consider including mentor as a fixed effect in the model.

Primary analyses Primary analyses will focus on estimation of efficacy as determined by (1) change in quality of life and (2) change in self-management. Estimates of effect sizes for outcome variables will be reported as point estimates (mean differences between pre-post measures, as appropriate) and interval estimates (95% CI) with two-sided p values denoting statistical significance to provide an indication of the presence of a clinically important treatment effect [99, 100]. A p value of 0.05 will be considered statistically significant. After studying the distributions of baseline characteristics, we will use a generalized, linear, mixed model, regression model to determine if the intervention will produce a greater change in the main outcomes from baseline. This model will include time, treatment (along with their interaction), and covariates (including the amount of intervention received, demographic factors, medications, coping, depression, stress, anxiety, health literacy, trust, and social support) as fixed effects. Using the amount of intervention, which is measured as an aggregate number of sessions completed or hours of interaction, as a

covariate would allow us to study the dose response. In the generalized linear mixed model, we will use different link functions depending on the assumed distribution of the response variable. For binary outcomes, we will use logit link and for count outcomes we will use log link under a Poisson or negative binomial distribution. For example, for a given quality-of -life variable, HRQOL, measured at baseline, month 3, and month 6, we will include intervention group, time, and time × intervention as the primary independent variables in the basic (unadjusted) model, and covariates that are not balanced at randomization (or a propensity score based on these covariates) will be added in the subsequent (adjusted) model to adjust for the possible confounding effect of these variables. Unadjusted and covariate-adjusted least squares means for each outcome variable will be compared at the primary time point (month 12) and at intermediate secondary time point (month 6) using appropriate model contrasts and the Tukey-Kramer adjustment for multiple comparisons for the secondary time points. These contrast comparisons, along with corresponding 95% CI, will provide estimates of the difference in outcome means (effect sizes) for the hypothesized comparisons.

Mid-point analyses In an effort to protect both mentors and mentees from potential deleterious effects of mentoring, a mid-point (interim) analysis will be undertaken to assess safety using 3-month post-intervention data. If mentored participants have worsened beyond a threshold, we will stop the trial for ethical concerns. For instance, the trial will be terminated if the lower confidence limits based on the 95% confidence interval at the midpoint, for any one of the variables, namely depression, anxiety, and/or disease activity, is larger than 50% compared with the baseline measure. Similarly, mentors will be monitored for depression, anxiety, and disease activity and if a worsening trajectory is observed at mid-point analyses, they will be removed from the study.

Cost-effectiveness analyses (CEA) The cost of the intervention will be compared to the outcomes of the intervention 12 months post-baseline. In order to compare with previous lupus studies [101, 102], Quality-adjusted life years (QALYS) will be calculated for intervention and control groups based on the Short Form 6D (SF-6D). The SF-6D permits the calculation of QALYs by estimating a preference-based single index measure for health from SF-36 data using general population values (https://www.sheffield.ac.uk/scharr/sections/heds/mvh/sf-6d). The SF-6D will be measured 12 months post-baseline. The calculation will be based on an established peer reviewed method [103]. Measuring QALYS gained relative to cost of the intervention is the preferred outcome method of the American College of Physicians [104].

Using QALYs, the incremental cost-effectiveness ratio (ICR) can be calculated as:

$(QALY_{intervention}\text{-}QALY_{control})/(Cost_{intervention}\text{-}Cost_{Control})$. The ICR can then be compared with the ICRs for previous lupus interventions. In addition to the main cost-effectiveness outcome of the QALY and the ICR, costs of the intervention can be compared with any changes in MUSC health services utilization costs for emergency department and inpatient and outpatient care for the intervention relative to the control group. In addition to a comparison of intervention cost with average difference between MUSC costs for intervention and control group 12 months post-baseline, generalized linear cost models can be estimated to examine the association of the treatment with MUSC health services costs while adjusting for patient demographics (age, gender, race/ethnicity, comorbidities) and clinical outcomes. In addition we will estimate the impact on work loss and income by estimating the changes in days lost to illness and income based on values provided by participants. We will use the year for which the hospital charge and income data are reported and adjust for inflation as appropriate using the US Department of Labor Consumer Price Index. Inpatient and outpatient MUSC costs will be compared separately and together between the control and intervention groups. Bootstrapping methods will be used to conduct sensitivity analyses for all cost models. Sensitivity analysis will be performed by estimating a separate MUSC health services cost model while adjusting for each clinical outcome to insure robust results on the marginal effect of the treatment on MUSC health services costs. Park tests will be conducted to determine the best fit for the cost data in specifying the generalized linear model. In the event of many zero values, a two-part model will be used to first examine the association of the treatment with the likelihood of any MUSC costs and then the conditional generalized linear cost model, conditional on having non-zero MUSC cost value for the patient. The ICR will be reported as a single ratio with no uncertainty attached to it. Therefore, standard statistical characteristics such as confidence intervals or hypothesis test to compare it to an a priori ICR from another study are not applicable. We will calculate the ICR and a clinically relevant interpretation of the outcome will be provided similar to other studies reported in the literature [105, 106].

Discussion

This study will test a culturally tailored intervention that promotes better understanding and management of a chronic condition by engaging individuals as active participants in their own health, in an effort to prevent

illness and promote health. This project is designed with the long-term goal of improving disease self-management and quality of life, and decreasing indicators of disease activity among African American patients with SLE and African American women suffering from other chronic illnesses. Specifically, this study is culturally tailored to the unique needs of African American women with SLE, will pair mentees with mentors who are race, gender, and SES concordant to facilitate bonding and social support, and will use peer mentors who are considered competent in the management of their condition in order to provide modeling and reinforcement to participants. It will be the first study to test peer mentorship as an alternative strategy to improve outcomes in a high-risk population with SLE. Given the success of the peer mentoring approach in other chronic conditions that disproportionately impact minorities, and its responsiveness to the needs of this unique population, this intervention is likely to result in health improvements that have not been attainable with other interventions and serve as a sustainable solution to persistent disparities in this population.

Trial status

The study started in September 2018. Permission has been granted by the Institutional Review Board (IRB) of the Medical University of South Carolina to start including participants, and the first wave of research participants are expected to be recruited by February 2019. At this time, recruitment and baseline data collection are in progress, and we expect the main RCT results to be published at the end of 2023.

Additional file

Additional file 1: SPIRIT checklist. (DOC 122 kb)

Abbreviations

ACR: American College of Rheumatology; AHI: Arthritis Helplessness Index; AIMS: Arthritis Impact Measurement Scales; AKA: Applied knowledge assessment; ASMP: Arthritis self-management program; BILD: Brief Index of Lupus Damage; CCCR: Core Center for Clinical Research; CDMP: Chronic disease self-management program; FACIT-F: Functional Assessment of Chronic Illness Therapy-Fatigue; GAD: General anxiety disorder; HRQOL: Health-related quality of life; ICR: Incremental cost-effectiveness ratio; IRB: Institutional Review Board; LUP-QOL: Lupus Quality of Life Questionnaire; MAR: Missing at random; MOS: Medical outcomes study; MSDRG: Medicare diagnosis related group; MTHCSS: Multidimensional Trust in Health Care Systems Scale; MUSC: Medical University of South Carolina; NRS: Numerical rating scale; PALS: Peer approaches to lupus self-management; PAM: Patient Activation Measure; PGA: Patient Global Assessment; PHQ: Patient Health Questionnaire; PI: Principal Investigator; PSS: Perceived stress scale; QALYS: Quality-adjusted life years; SF-36: Short Form 36 Health Survey; SLAQ: Systemic Lupus Activity Questionnaire; SLE: Systemic lupus erythematosus; SLEDAI: Systemic Lupus Disease Activity Index; SLESH: Systemic lupus erythematosus self-help; UCLA: University of California at Los Angeles

Acknowledgements
Not applicable.

Authors' contributions
EMW is the principal investigator and LE and JO are senior co-investigators who assisted with conceptual development. VR developed the statistical analysis plan and CD developed the plan for cost-effectiveness analyses (CEA). TDF was involved in intervention development, implementation, evaluation, data analysis, and manuscript writing for the PALS pilot study. HJ developed educational content and JR assisted with development of the mentor training manual and strategy. All authors read and approved the final version for publication.

Funding
Research reported in this publication was supported by the National Institute of Nursing Research of the National Institutes of Health under Award Number R01NR017892. The content is solely the responsibility of the authors and does not necessarily represent the official views of the National Institutes of Health.

Availability of data and materials
Not applicable.

Ethics approval and consent to participate
The study protocol has been approved by the IRB of the Medical University of South Carolina (MUSC) (Pro00080875). Informed consent will be obtained from all study participants prior to their participation in the study. Any changes to the study procedures will first be proposed to the IRB.

Consent for publication
Not applicable.

Competing interests
The authors declare that they have no competing interests.

Author details
[1]Department of Public Health Sciences, Medical University of South Carolina, 135 Cannon Street, Suite CS303D, Charleston, SC 29425, USA. [2]Department of Medicine, Medical College of Wisconsin, 8701 Watertown Plank Road, Milwaukee, WI 53226, USA. [3]Division of Rheumatology and Immunology, Medical University of South Carolina, 96 Jonathan Lucas St, Charleston, SC 29425, USA. [4]Rheumatology Section, Ralph H. Johnson VA Medical Center, 109 Bee Street, Charleston, SC 29401, USA. [5]Heath Economics Resource Center (HERC), VA Palo Alto Medical Care System, 795 Willow Road (152 MPD), Menlo Park, CA 94025, USA. [6]Lupus Columbia SC, 1900 Kathleen Drive, Columbia, SC 29210, USA. [7]Department of Social Work Programs, Hospital for Special Surgery, 535 East 70th Street, New York, NY 10021, USA.

Received: 11 February 2019 Accepted: 16 July 2019
Published online: 23 August 2019

References
1. Rahman A, Isenberg D. Systemic lupus erythematosus. New Engl J Med. 2008;358(9):929–39.
2. Pons-Estel G, Ugarte-Gil M, Alarcón G. Epidemiology of systemic lupus erythematosus. Expert Rev Clin Immu. 2017;13(8):799–814.
3. Giffords E. Understanding and managing systemic lupus erythematosus (SLE). J Soc Work Health Care. 2003;37(4):57–72.
4. Sehlo M, Bahlas S. Perceived illness stigma is associated with depression in female patients with systemic lupus erythematosus. J Psychosom Res. 2013;74(3):248–51.
5. Hanly J, Su L, Urowitz M, et al. Mood disorders in systemic lupus erythematosus: results from an international inception cohort study. Arthritis Rheumatol. 2015;67(7):1837–47.
6. Kulczycka L, Sysa-Jędrzejowska A, Robak E. The influence of clinical manifestations and treatment on satisfaction with life together with positive and negative emotions in systemic lupus erythematosus patients. Acta Dermatovenerol Croat. 2011;19(1):6–12.
7. Jolly M. How does quality of life of patients with systemic lupus erythematosus compare with that of other common chronic illnesses? J Rheumatol. 2005;32(9):1706–8.

8. Beckerman N. Living with lupus: a qualitative report. Soc Work Health Care. 2011;50(4):330–43.
9. McElhone K, Abbott J, Gray J, Williams A, Teh L-S. Patient perspective of systemic lupus erythematosus in relation to health related quality of life concepts. A qualitative study. Lupus. 2010;19(14):1640–7.
10. Macejová Z, Záriková M, Oetterová M. Systemic lupus erythematosus–disease impact on patients. Cent Eur J Public Health. 2013;21(3):171–3.
11. Campbell RJ, Cooper G, Gilkeson G. The impact of systemic lupus erythematosus on employment. J Rheumatol. 2009;36(11):2470–5.
12. Williams E, Bruner L, Adkins A, Vrana C, Logan A, Kamen D, et al. I too, am America: a review of research on systemic lupus erythematosus (SLE) in African Americans. Lupus Sci Med. 2015;3(1):E000144.
13. Fernández M, Alarcón G, Calvo-Alén J, et al. A multiethnic, multicenter cohort of patients with systemic lupus erythematosus (SLE) as a model for the study of ethnic disparities in SLE. Arthritis and Rheum. 2007;57(4):576–84.
14. Lau C, Yin G, Mok M. Ethnic and geographical differences in systemic lupus erythematosus: an overview. Lupus. 2006;15(11):715–9.
15. Ow M, Ho P, Thumboo J, Wee H. Factors associated with health services utilization in patients with systemic lupus erythematosus: a systematic review. Clin Exp Rheumatol. 2010;28(6):892–904.
16. Cooper G, Parks C, Treadwell E, St. Clair E, Gilkeson G, Cohen P, et al. Differences by race, sex and age in the clinical immunologic features of recently diagnosed systemic lupus erythematosus patients in the southeastern United States. Lupus. 2002;11(3):161.
17. Alarcon G, Beasley T, Roseman J. Ethnic disparities in health and disease: the need to account for ancestral admixture when estimating the genetic contribution to both (LUMINA XXVI). Lupus. 2005;14(10):867–8.
18. Williams E, Egede L, Oates J. Effective self-management interventions for patients with lupus: potential impact of peer mentoring. Am J Med Sci. 2017;353(6):580–92.
19. Lorig K, Ritter P, Plant K. A disease-specific self-help program compared with a generalized chronic disease self-help program for arthritis patients. Arthritis Rheum. 2005;53(6):950–7.
20. Lorig K, Ritter P, Laurent D, Fries J. Long-term randomized controlled trials of tailored-print and small-group arthritis self-management interventions. Med Care. 2004;42(4):346–54.
21. Lorig K, Ritter P, Laurent D, Plant K. The Internet-based arthritis self-management program: a one-year randomized trial for patients with arthritis or fibromyalgia. Arthritis Rheum. 2008;59(7):1009–17.
22. Greco C, Rudy T, Manzi S. Effects of a stress-education program on psychological function, pain, and physical function of systemic lupus erythematosus patients: a randomized controlled trial. Arthritis Rheum. 2004;51(4):625–34.
23. Edworthy S, Dobkin P, Clarke A, Da Costa D, Dritsa M, Fortin P, et al. Group psychotherapy reduces illness intrusiveness in systemic lupus erythematosus. J Rheumatol. 2003;30(5):1011–6.
24. Brady T, Kruger J, Helmick C, Callahan L, Boutaugh M. Intervention programs for arthritis and other rheumatic diseases. Health Educ Behav. 2003;30(1):44–63.
25. Drenkard C, Bao G, Dennis G, Kan H, Jhingran P, Molta C, et al. Burden of systemic lupus erythematosus on employment and work productivity: data from a large cohort in the southeastern United States. Arthritis Care Res. 2014;66(6):878–87.
26. Wallace R. Systemic lupus erythematosus in African-American women: cognitive physiological modules, autoimmune disease, and structured psychosocial stress. Adv Complex Syst. 2003;6(4):599–629.
27. Barnado A, Wheless L, Meyer A, Gilkeson GL, Kamen D. Quality of life in patients with systemic lupus erythematosus (SLE) compared with related controls within a unique African American population. Lupus. 2012;21(5):563–9.
28. Chae D, Drenkard C, Lewis T, Lim S. Discrimination and cumulative disease damage among African American women with systemic lupus erythematosus. Am J Public Health. 2015;105(10):2099–107.
29. Feldman C, Bermas B, Zibit M, Fraser P, Todd D, Fortin P, et al. Designing an intervention for women with systemic lupus erythematosus from medically underserved areas to improve care: a qualitative study. Lupus. 2013;22(1):52–62.
30. Sutanto B, Singh-Grewal D, McNeil H, O'Neill S, Craig J, Jones J, et al. Experiences and perspectives of adults living with systemic lupus erythematosus: thematic synthesis of qualitative studies. Arthritis Care Res. 2013;65(11):1752–65.
31. Danoff-Burg S, Friedberg F. Unmet needs of patients with systemic lupus erythematosus. Behav Med. 2009;35(1):5–13.
32. Martin L, Williams S, Haskard K, DiMatteo M. The challenge of patient adherence. Ther Clin Risk Manag. 2005;1(3):189–99.
33. Julian L, Yelin E, Yazdany J, Panopalis P, Trupin L, Criswell L, et al. Depression, medication adherence, and service utilization in systemic lupus erythematosus. Arthritis Rheum. 2009;61(2):240–6.
34. de Achaval S, Suarez-Almazor M. Improving treatment adherence in patients with rheumatologic disease. J Musculoskelet Med. 2010;27(10):1691476.
35. Korbet S, Schwartz M, Evans J, Lewis E. Severe lupus nephritis: racial differences in presentation and outcome. J Am Soc Nephrol. 2007;18(1):244–54.
36. Moses N, Wiggers J, Nicholas C, Cockburn J. Prevalence and correlates of perceived unmet needs of people with systemic lupus erythematosus. Patient Educ Couns. 2005;57(1):30–8.
37. Law G, Pope J, Lalani S, Silverman E, Cooper G, Fortin P, et al. Barriers To healthcare in a multiethnic cohort of systemic lupus erythematosus (SLE) patients: patient and physician perceptions. Rom J Rheum. 2010;19(1):12–9.
38. Mosley-Williams A, Lumley M, Gillis M, Leisen J. D G. Barriers to treatment adherence among African-American and white women with systemic lupus erythematosus. Arthritis Rheum. 2002;47(6):630–8.
39. Heisler M. Different models to mobilize peer support to improve diabetes self-management and clinical outcomes: evidence, logistics, evaluation considerations and needs for future research. Fam Pract. 2010;27(Suppl 1):i23–32.
40. Heisler M. Building peer support programs to manage chronic disease: seven models for success. Oakland: California Healthcare Foundation; 2006.
41. Rotheram-Borus M, Richter L, van Heerden A, van Rooyen H, Tomlinson M, Harwood J, et al. A cluster randomized controlled trial evaluating the efficacy of peer mentors to support South African women living with HIV and their infants. PLoS One. 2014;9(1):e84867.
42. Jerson B, D'Urso C, Arnon R, Miloh T, Iyer K, Kerkar N, et al. Adolescent transplant recipients as peer mentors: a program to improve self-management and health-related quality of life. Pediatr Transplant. 2013;17(7):612–20.
43. Tracy K, Burton M, Nich C, Rounsaville B. Utilizing peer mentorship to engage high recidivism substance-abusing patients in treatment. Am J Drug Alcohol Abuse. 2011;37(6):525–31.
44. Anderson A, Damio G, Chapman D, Perez-Escamilla R. Differential response to an exclusive breastfeeding peer counseling intervention: the role of ethnicity. J Hum Lact. 2007;23(1):16–23.
45. Spencer R, Bower J, Kirk S, Hancock FC. Peer mentoring is associated with positive change in physical activity and aerobic fitness of grades 4, 5, and 6 students in the heart healthy kids program. Health Promot Pract. 2014;15(6):803–11.
46. Thomas R, Lorenzetti D, Spragins W. Systematic review of mentoring to prevent or reduce tobacco use by adolescents. Acad Pediatr. 2013;13(4):300–7.
47. Dorgo S, Robinson K, Bader J. The effectiveness of a peer-mentored older adult fitness program on perceived physical, mental, and social function. J Am Acad Nurse Pract. 2009;21(2):116–22.
48. Eskicioglu P, Halas J, Senechal M, Wood L, McKay E, Villeneuve S, et al. Peer mentoring for type 2 diabetes prevention in first nations children. Pediatrics. 2014;133(6):e1624–e31.
49. Keyserling T, Samuel-Hodge C, Ammerman A, Ainsworth B, Henriquez-Roldan C, Elasy T, et al. A randomized trial of an intervention to improve self-care behaviors of African-American women with type 2 diabetes: impact on physical activity. Diabetes Care. 2002;25(9):1576–83.
50. Heisler M, Piette J. "I help you, and you help me": facilitated telephone peer support among patients with diabetes. Diabetes Educ. 2005;31:869–79.
51. Sazlina S, Browning C, Yasin S. Effectiveness of personalized feedback alone or combined with peer support to improve physical activity in sedentary older Malays with type 2 diabetes: a randomized controlled trial. Front Public Health. 2015;3:178.
52. Knox L, Huff J, Graham D, Henry M, Bracho A, Henderson C, et al. What peer mentoring adds to already good patient care: implementing the Carpeta Roja peer mentoring program in a well-resourced health care system. Ann Fam Med. 2015;13(Suppl 1):S59–565.
53. Woodbury M, Botros M, Kuhnke J, Greene J. Evaluation of a peer-led self-management education programme PEP talk: diabetes, healthy feet and you. Int Wound J. 2013;10(6):703–11.
54. Philis-Tsimikas A, Fortmann A, Lleva-Ocana L, Walker C, Gallo L. Peerled diabetes education programs in high-risk Mexican Americans improve glycemic control compared with standard approaches: a Project Dulce promotora randomized trial. Diabetes Care. 2011;34:1926–31.
55. Long J, Jahnle E, Richardson D, Loewenstein G, Volpp K. Peer mentoring and financial incentives to improve glucose control in African American veterans: a randomized trial. Ann Intern Med. 2012;156(6):416–24.

56. Williams E, Hyer M, Voronca D, Ramakrishnan V, Faith T, Gebregziabher M, et al. Peer-to-peer mentoring for African American women with lupus: a feasibility pilot. Arthritis Care Res. 2018;70(6):908–17.
57. Williams E, Hyer J, Ramakrishnan V, Faith T, Egede L, Oates J, et al. Cytokine balance and behavioral intervention; findings from the Peer Approaches to Lupus Self-Management (PALS) project. Hum Immunol. 2017;78(9):574–81.
58. Bellg A, Borrelli B, Resnick B, Hecht J, Minicucci D, Ory M, et al. Enhancing treatment fidelity in health behavior change studies: best practices and recommendations from the NIH Behavior Change Consortium. Health Psychol. 2004;23(5):443–51.
59. Hochberg M. Updating the American College of Rheumatology revised criteria for the classification of systemic lupus erythematosus (letter). Arthritis Rheum. 1997;40(9):1725.
60. Peterson M, Horton R, Engelhard E, Lockshin M, Abramson T. Effect of counselor training on skills development and psychosocial status of volunteers with systemic lupus erythematosus. Arthritis Care Res. 1993;6(1):38–44.
61. Allen L, Tsao J, Hayes L, Zeltzer L. Peer mentorship to promote effective pain management in adolescents: study protocol for a randomised controlled trial. Trials. 2011;12:132.
62. Horton R, Peterson M, Powell S, Engelhard E, Paget S. Users evaluate LupusLine, a telephone peer counseling service. Arthritis Care Res. 1997; 10(4):257–63.
63. Sandhu S, Veinot P, Embuldeniya G, Brooks S, Sale J, Huang S, et al. Peer-to-peer mentoring for individuals with early inflammatory arthritis: feasibility pilot. BMJ Open. 2013;3(3):e002267.
64. Kang M, Ragan B, Park J. Issues in outcomes research: an overview of randomization techniques for clinical trials. J Athl Train. 2008;43(2):215–21.
65. Arthritis Foundation. The Systemic Lupus Erythematosus Self-Help Course: program guidelines and procedures manual: Atlanta: Arthritis Foundation; 1987.
66. Williams E, Penfield M, Kamen D, Oates J. An intervention to reduce psychosocial and biological indicators of stress in african american lupus patients: the Balancing Lupus Experiences with Stress Strategies Study. Open J Prev Med. 2014;4(1):22–31.
67. Chinman M, McCarthy S, Mitchell-Miland C, Daniels K, Youk A, Edelen M. Early stages of development of a peer specialist fidelity measure. Psychiatr Rehabil J. 2016;39(3):256–65.
68. MUSC Office of Public Relations. Patients, families with lupus receive support. The Catalyst. 2011 Friday, November 25, 2011.
69. Queen Quet. St. Helena Island, SC 2017. [cited 2017]. Available from: https://gullahgeecheenation.com/2017/05/18/lupus-awareness-day-the-gullahgeechee-way/.
70. Flournoy-Floyd M, Ortiz K, Oates J, Egede L, Williams E. "We Would Still Find Things to Talk About": assessment of mentor perspectives in a systemic lupus erythematosus intervention (Peer Approaches to Lupus Self-management-PALS), empowering SLE patients. J Natl Med Assoc. 2018;110(2):182–9.
71. Egede L, Strom J, Durkalski V, Mauldin P, Moran W. Rationale and design: telephone-delivered behavioral skills interventions for blacks with type 2 diabetes. Trials. 2010;11:35.
72. Williams J, Lynch C, Knapp R, Egede L. Technology-Intensified Diabetes Education Study (TIDES) in African Americans with type 2 diabetes: study protocol for a randomized controlled trial. Trials. 2014;15:460.
73. Matthews B, Baker F, Hann D, Denniston M, Smith T. Health status and life satisfaction among breast cancer survivor peer support volunteers. Psychooncology. 2002;11(3):199–211.
74. Stewart A, Hays R, Ware J. Measuring functioning and well-being: the Medical Outcomes Study approach. In: Stewart A, Ware J, editors. Health Perceptions, Energy/Fatigue, and Health Distress Measures. Durham: Duke University Press; 1991.
75. Webster K, Cella D, Yost K. The Functional Assessment of Chronic Illness Therapy (FACIT) measurement system: properties, application, and interpretation. Health Qual Life Out. 2003;16(1):79.
76. Toloza S, Jolly M, Alarcón G. Quality-of-life measurements in multiethnic patients with systemic lupus erythematosus: cross-cultural issues. Curr Rheumatol Rep. 2010;12(4):237–49.
77. Hibbard J, Stockard J, Mahoney E, Tusler M. Development of the Patient Activation Measure (PAM): conceptualizing and measuring activation in patients and consumers. Health Serv Res. 2004;39(4 Pt 1):1005–26.
78. Hibbard J, Mahoney E, Stockard J, Tusler M. Development and testing of a short form of the patient activation measure. Health Serv Res. 2005;40(6 Pt 1):1918–30.
79. Lorig K, Stewart A, Ritter P, Gonzalez V, Laurent D, Lynch J. Outcome measures for health educaiton and other health care interventions. Thousand Oaks: Sage Publications; 1996.
80. Lorig K, Sobel D, Ritter P, Laurent D, Hobbs M. Effect of a self-managment program on patients with chronic disease. Effective Clinical Practice. 2001; 4(6):256–62.
81. Ortiz K, Flournoy-Floyd M, Williams E. Systemic Lupus Erythematosus Observations of Travel Burden (SLEOTB): a qualitative inquiry. Int J Rheum Dis. 2015;18(7):751–60.
82. Williams EM, Bruner L, Ortiz K, Zhang J, Zhou J, Kamen D. The Systemic Lupus Erythematosus Travel Burden Survey: baseline data among a South Carolina cohort. BMC Res Notes. 2016;9:246.
83. Hays R, Cunningham W, Sherbourne C, Wilson I, Wu A, Cleary P, et al. Health-related quality of life in patients with human immunodeficiency virus infection in the United States: results from the HIV Cost and Services Utilization Study. Am J Med. 2000;108(9):714–22.
84. Lorig K, Chastain R, Ung E, Shoor S, Holman H. Development and evaluation of a scale to measure perceived self-efficacy in people with arthritis. Arthritis Rheum. 1989;32(1):37–44.
85. Kroenke K, Spitzer R. The PHQ-9: a new depression and diagnostic severity measure. Psychiat Ann. 2002;32(9):509–21.
86. Spitzer R, Kroenke K, Williams J, Löwe B. A brief measure for assessing generalized anxiety disorder: the GAD-7. Arch Intern Med. 2006;166(10):1092–7.
87. Cohen S, Kamarck T, Mermelstein R. A global measure of perceived stress. J Health Soc Behav. 1983;24(4):385–96.
88. Baker D, Williams M, Parker R, Gazmararian J, Nurse J. Development of a brief test to measure functional health literacy. Patient Educ Couns. 1999; 38(1):33–42.
89. Sherbourne C, Stewart A. The MOS social support survey. Soc Sci Med. 1991; 32(6):705–14.
90. Egede L, Ellis C. Development and testing of the Multidimensional Trust in Health Care Systems Scale. J Gen Intern Med. 2008;23(6):808–15.
91. Karlson E, Daltroy L, Rivest C, et al. Validation of a systemic lupus activity questionnaire (SLAQ) for population studies. Lupus. 2003;12(4):280–6.
92. Bombardier C, Gladman D, Urowitz M, Caron D, Chang C, Austin A, et al. Derivation of the SLEDAI. A disease activity index for lupus patients. Arthritis Rheum. 1992;35(5):630–40.
93. American College of Rheumatology Ad Hoc Committee on Systemic Lupus Erythematosus Response Criteria. The American College of Rheumatology response criteria for systemic lupus erythematosus clinical trials: measures of overall disease activity. Arthritis Rheum. 2004;50(11):3418–26.
94. Lu Q, You J, Man J, Loh A, Young L. Evaluating a culturally tailored peer-mentoring and education pilot intervention among Chinese breast cancer survivors using a mixed-methods approach. Oncol Nurs Forum. 2014;41(6):629–37.
95. Collings R, Swanson V, Watkins R. The impact of peer mentoring on levels of student wellbeing, integration and retention: a controlled comparative evaluation of residential students in UK higher education. High Educ. 2014; 68(6):927–42.
96. Chung M, Moser D, Lennie T, Frazier S. Perceived social support predicted quality of life in patients with heart failure, but the effect is mediated by depressive symptoms. Qual Life Res. 2013;22(7):1555–63.
97. Merianos A, King K, Vidourek R, Nabors L. Mentoring and peer-led interventions to improve quality of life outcomes among adolescents with chronic illnesses. Appl Res Qual Life. 2016;11(3):1009–23.
98. Keefe R, Kraemer H, Epstein R, Frank E, Haynes G, Laughren T, et al. Defining a clinically meaningful effect for the design and interpretation of randomized controlled trials. Innov Clin Neurosci. 2013;10(5–6 Suppl A):4S–19S.
99. Donner A. Sample size requirements for the comparison of two or more coefficients of interobserver agreement. Stat Med. 1998;17(10):1157.
100. Eldridge S, Ashby D, Kerry S. Sample size for cluster randomized trials: effect of coefficient of variation of cluster size and analysis method. Int J Epidemiol. 2006;35(5):1292–300.
101. Wilson E, Jayne R, Dellow E. Fordham. The cost-effectiveness of mycophenolate mofetil as firstline therapy in active lupus nephritis. Rheumatology. 2007;46(7):1096–101.
102. Pierotti F, Palla I, Treur M, Pippo L, Turchetti G. Assessment of the economic impact of belimumab for the treatment of systemic lupus erythematosus in the Italian setting: a cost-effectiveness analysis. PLoS One. 2015;10(10): e0140843.

103. Nichol M, Sengupta N, Globe D. Evaluating quality-adjusted life years: estimation of the health utility index (HUI2) from the SF −36. Med Decis Mak. 2001;21(2):105–12.
104. Owens D, Qaseem A, Chou R, Shekelle P. High-value, cost-conscious health care: concepts for clinicians to evaluate the benefits, harms, and costs of medical interventions. Ann Intern Med. 2011;154(3):174–80.
105. Dixon P, Hollinghurst S, Edwards L, Thomas C, Foster A, Davies B, et al. Cost-effectiveness of telehealth for patients with depression: evidence from the Healthlines randomised controlled trial. BJPsych Open. 2016;2:262–9.
106. Pyne J, Fortney J, Tripathi S, Maciejewski M, Edlund M, Williams D. Cost-effectiveness analysis of a rural telemedicine collaborative care intervention for depression. Arch Gen Psychiatry. 2010;67(8):812–21.

Publisher's Note

Springer Nature remains neutral with regard to jurisdictional claims in published maps and institutional affiliations.

Ready to submit your research? Choose BMC and benefit from:

- fast, convenient online submission
- thorough peer review by experienced researchers in your field
- rapid publication on acceptance
- support for research data, including large and complex data types
- gold Open Access which fosters wider collaboration and increased citations
- maximum visibility for your research: over 100M website views per year

At BMC, research is always in progress.

Learn more biomedcentral.com/submissions

BMC
